# The Use of Golf Carts as On-Course Transportation During Competitive Golf Events: A Scoping Review

**DOI:** 10.1007/s40279-025-02354-8

**Published:** 2025-11-18

**Authors:** Andrew J. Hall, Chris Bishop, Amy O’Donnell, Tony Bennett, Graeme L. Close, Wimpie Du Plessis, Andrew D. Murray

**Affiliations:** 1 School of Medicine, University of St Andrews, North Haugh, St Andrews, Fife KY16 9TF UK; 2 Sport and Exercise, University of Edinburgh, Edinburgh, UK; 3 European Tour Performance Institute, European Tour Group, Virginia Water, UK; 4 London Sports Institute, University of Middlesex, London, UK; 5 Research Institute for Sport and Exercise Sciences, Liverpool John Moores University, Liverpool, UK; 6 Ladies European Tour Performance Institute, Ladies European Tour, Denham, UK; 7 Disability and Inclusion, International Golf Federation, Lausanne, Switzerland; 8 Presidents Office, EDGA (Formerly European Disabled Golf Association), Wassenaar, Netherlands; 9 Medical and Scientific Department, Sunshine Tour, Johannesburg, South Africa; 10 Medical and Scientific Department, The R&A, St Andrews, UK

## Abstract

**Background:**

Golf is enjoyed by over 108 million people globally, and has established elite female, male and disability competitive schedules. The use of golf carts during elite competition is a topic of discussion regarding the principles of inclusivity and competitive fairness with important scientific, legal and ethical considerations.

**Objective:**

The primary aim was to evaluate the evidence relating to riding a golf cart in comparison to walking the golf course, pertaining specifically to: (1) inclusivity and safety, and (2) competitive fairness. The secondary aim was to identify knowledge gaps and research priorities.

**Methods:**

We conducted a scoping review and the search strategy was applied to the following databases, and articles were extracted by independent reviewers from PubMed, Web of Science, Scopus and ProQuest Central. Grey literature was examined using Google Advanced Search. A five-stage scoping review methodology followed the Preferred Reporting Items for Systematic Reviews and Meta-Analyses extension for Scoping Reviews (PRISMA-ScR). Articles were collated using an online tool (Covidence) and evaluated against pre-determined criteria. Data from included studies were collated to facilitate descriptive and thematic analysis.

**Results:**

Of the 879 studies identified, 72 were included for analysis. Seven themes emerged: physical demands, inclusion and accessibility, cognitive demands, legal and ethical, nutrition and hydration, competitive fairness, and safety and injury risk. Golf carts promote accessibility, particularly for individuals with disabilities. Safety concerns predominantly arise in non-elite settings, with incidents linked to operator error, poor safety governance and use on public highways. There is compelling evidence demonstrating that in-round riding of golf carts reduces the physical demands of golf, with users expending less energy and covering shorter distances, although effects on performance is uncertain. Golf cart use may mitigate the environmental aspects of golf, including the effects of hot/humid weather or hilly topography.

**Conclusions:**

Golf carts can increase inclusivity for golfers and enable clubs and organisations to meet their ethical and legal requirements around accessibility. However, riding golf carts may diminish some of the health benefits of golf, and present a risk of injury and practical challenges. In the competitive setting, golf cart use reduces the physical demands of the game, affecting the environmental challenges of variable weather and terrain. It is biologically plausible and likely that this would offer a performance advantage in some circumstances. More research is needed to guide the fair use of this enabling technology within clearly defined contexts by: (1) evaluating the performance deficits associated with medical issues in which the use of golf carts might be considered permissible, and (2) evaluating the magnitude and nature of competitive advantages experienced by golf cart riders.

**Supplementary Information:**

The online version contains supplementary material available at 10.1007/s40279-025-02354-8.

## Key Points

**Table:** 

Golf carts support greater accessibility by allowing individuals with mobility limitations to engage in the sport. Although the safety concerns related to golf cart use are well established, they largely pertain to recreational settings rather than elite or professional competition environments.
Riding a golf cart lessens the physical demands of play, helps manage fatigue and reduces environmental strain. This may mitigate the effects of disability associated with some medical conditions (performance enabling), but in some circumstances could provide an additional advantage (performance enhancing).
Evidence-informed and consistently applied policies are essential to ensure both inclusivity and fairness in competition, particularly for athletes requesting medical exemptions.

## Introduction

Golf is a popular and inclusive sport played recreationally by 108 million people of all ages globally, and its economic value exceeds $225 billion annually [1, 2]. Golf can provide health-enhancing physical activity benefits, and is associated with long-term physical and mental health improvements [3, 4]. Professional golf is a global industry, and golfers may be eligible for elite golf “leagues” or “tours”, such as the Asian Tour, DP World Tour (DPWT), Golf 4 Disabled (G4D) Tour, Ladies European Tour (LET), Ladies Professional Golf Association (LPGA) Tour, LIV Golf and Professional Golf Association (PGA) Tour. Typically, a round of golf involves 3–6 h of low-to-moderate intensity exercise resulting in many health-related benefits for the players. In addition, these health benefits also extend to support staff (including caddies) and spectators who experience notable physical activity when they attend events [5–7]. Over 10 million spectators attend professional golf events annually [8].

Golf organisations including the global governing bodies: (i) The R&A, and (ii) the United States Golf Association (USGA) continue to promote increasing inclusivity in the sport to maximise participation and the associated health and well-being benefits that golf can bring [4]. However, these and other organisations have also indicated a desire to maintain competitive fairness, with regard to their elite competitions [9, 10]. Further, legal and policy frameworks require that sports organisations make reasonable accommodations for disabled individuals, which may include access to golf carts [11, 12]. A landmark example is the US Supreme Court ruling in favour of professional golfer Casey Martin, whose rare condition (Klippel-Trénaunay syndrome) warranted golf cart use during PGA Tour events. This case established an important precedent, reinforcing the need to balance inclusion with the preservation of competitive integrity [13, 14]. However, the boundaries of what qualifies as a disability remain contested. Recent cases have sparked debate around the legitimacy of medical exemptions and raised questions about how to distinguish between disability, injury, poor conditioning and age-related decline [15, 16]. These ambiguities highlight the need for clearer evidence-based criteria and consistent policy to guide decisions in elite golf contexts.

The rules relating to on-course transportation (riding a golf cart vs walking the course) in elite golf are currently under discussion, and governing organisations are engaged in consultation regarding the use of transportation in elite competition. Inclusion, fairness (and to an extent) safety have been central issues in the discussion regarding allowing professional players to use golf carts, in particular for individuals that might otherwise be unable to participate because of injury or ill health or disability. Golf carts are permitted in competition for golfers with disabilities and in some senior golfer events, but before the riding of golf carts in professional competition beyond these specific settings can be considered, there is a requirement to evaluate whether their use provides players with a competitive advantage compared to those walking the course [17–19].

Factors related to on-course transportation that may influence performance include differences in physical workload, cognitive workloads, and exposure and response to environmental factors, such as extreme heat, humidity, high altitude, wind or heavy rain. Furthermore, there is a need to evaluate whether any such competitive advantage could be mitigated or eliminated to maintain competitive integrity. Consequently, the aim of this scoping review was to evaluate the current scientific literature relating to on-course transportation (riding of a golf cart) in comparison to no transportation (walking the course) when playing competitive golf. The primary objectives were to identify the main issues relating to: (i) inclusion and safety, and (ii) competitive fairness and performance. The secondary objectives were to identify knowledge gaps and research priorities related to on-course transportation in the sport.

## Methods

The present study adhered to an established five-stage scoping review methodology following the Preferred Reporting Items for Systematic Reviews and Meta-Analyses extension for Scoping Reviews (PRISMA-ScR), and was conducted in accordance with current best practice [20–23]. The methodological approach, research question, search strategy and analyses were conducted while including consultation between the author team and the scientific leads for key professional stakeholders (The International Golf Federation, The R&A, The Ladies European Tour Group, the European Tour Group, G4D and EDGA [formerly European Disabled Golf Association]).

### Stage 1: Identification of Research Questions

The following research questions were considered with respect to the use of on-course transportation (riding golf carts) compared to no on-course transport (walking):

1. What are the main issues relating to inclusion and safety in competitive golf?
2. What evidence exists regarding performance and competitive fairness in golf?

### Stage 2: Identification of Relevant Studies

The search inclusion and exclusion criteria were established through discussion between the author team and with relevant officials, who were considered to be experts in golf.

*Inclusion Criteria*

- Articles published between 1 January, 2000 and 28 October, 2024 (date searches were conducted).
- All ages and genders of human subjects.
- All levels of golf (including recreational, amateur competitive, elite/professional).
- Related to all forms of on-course golf (including but not limited to 18-hole, 9-hole, individual formats, team formats, stroke play, match play, single round and multi-round).
- Sources of information: original research (observational, experimental); reviews (including narrative, scoping, systematic); meta-analyses; guidelines; grey literature (e.g. ongoing registered research, academic dissertations, conference proceedings, annual reports).
- Only review articles or original research studies with significant regional or national coverage were included when considering studies that were specific to golf cart injuries (owing to the very large volume of studies published concerning golf cart accidents outside of a golf context).

*Exclusion Criteria*

- Full text unavailable.
- Non-English language articles, or articles without an available English translation.
- Unsupported opinion or comment (e.g. articles in magazines, blogs, journals or spoken commentary, presented without supporting data).

#### Search Strategies and Databases

An initial search of the literature was conducted using the PubMed and Google Scholar databases to identify relevant articles and themes related to the study questions. Text from titles and abstracts of appropriate articles were used to develop a set of search terms, and a formal search strategy was constructed and agreed through consensus by two authors (AJH and ADM).

Terms were applied to titles and abstracts using Boolean operators to combine key concepts and constructed into search strings formatted according to the requirements of each electronic database. These search terms included: golf, golf cart, buggy, transport, performance, scoring, score, ranking, competitive, advantage, disadvantage, injury, illness, health, disability, disabled, ability, access, inclusion, exclusion, demand, metabolic demand, physical, workload, fatigue, tiredness, golf course, terrain, topography, elevation, heat, cold, wind, rain, environment, fairness, competition, equality, benefit, energy expenditure, energy intake, caloric cost, metabolic cost, calorie, activity energy expenditure, metabolic expenditure, putting accuracy, putting performance, clubhead speed, swing speed, clubhead velocity, drive distance, driving accuracy, shot precision, shot accuracy, sweat rate, sweat, cardiovascular, cardiorespiratory, heart rate, actiheart monitor, pulse, cognitive function, decision making, cognitive-motor performance, executive function, concentration, attention, hydration, dehydration, euhydration.

The search strategy was applied to the following databases to extract relevant literature: PubMed, Web of Science, Scopus and ProQuest Central. Regarding exploration of the grey literature, a Google Scholar advanced search was utilised to search for relevant articles from the global organisations responsible for golf development that have academic operations (World Golf Foundation and The R&A), leading academic sports medicine journal websites and non-indexed golf-specific scientific publications (*International Journal of Golf Science*). The reference lists of all included studies were screened for any additional relevant articles.

### Stage 3: Study Selection

**Fig. 1:**
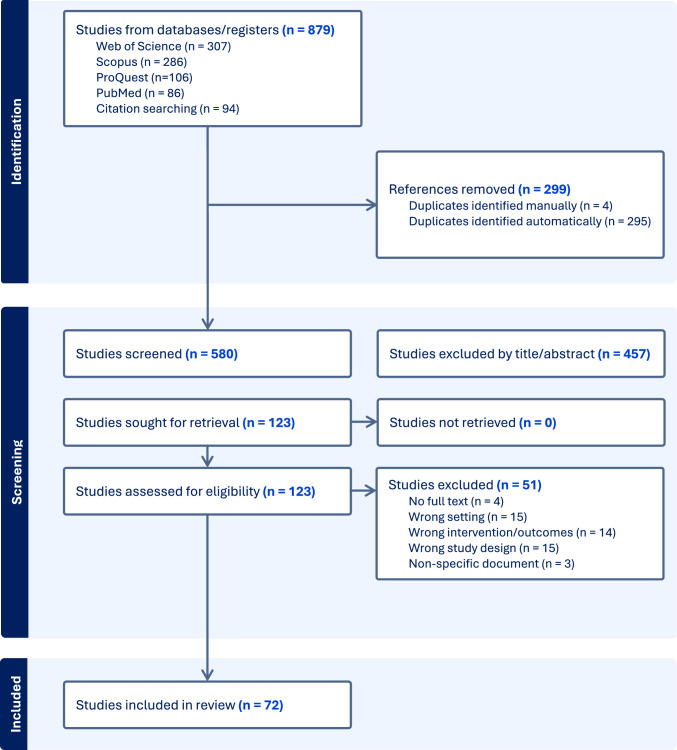
Preferred Reporting Items for Systematic Reviews and Meta-Analyses extension for Scoping Reviews (PRISMA-ScR) flow chart of the search process and included articles

Following a search of the relevant databases, all identified articles were collated using a dedicated collaborative review platform (Covidence [Covidence systematic review software; Veritas Health Innovation, Melbourne, VIC, Australia]) and duplicates subsequently removed. Titles and abstracts were screened by two reviewers independently (AH and AM) and assessed against the inclusion and exclusion criteria. Full-text articles were retrieved for all potentially relevant sources, and a detailed assessment against the inclusion and exclusion criteria was conducted by the lead reviewer (AJH). A secondary reviewer (ADM) completed the same process on 10% of the titles chosen at random sample from a list arranged alphabetically to avoid relevancy or recency bias. No disagreements between the two reviewers occurred; thus, no further discussions with a third reviewer were deemed necessary. The results of this search process were reported using a PRISMA-ScR flow diagram (Fig. 1).

### Stage 4: Extracting the Data

The search results were incorporated into data extraction/charting tables to facilitate the extraction of relevant data by the lead reviewer (AJH), who extracted 100% of the results. A secondary reviewer (ADM) reviewed 10% of the extracted data independently to ensure accuracy and consistency in the information being considered. Data extraction categories included: author, year of publication, source of publication (e.g. journal name), where the study was conducted, stated study aims, study population and sample size, methodology, outcome measures, context (relevance to golf), findings (relevant to the study questions), strengths and weaknesses.

### Stage 5: Collating, Summarising and Reporting the Results

Data were collated and summarised utilising:

1. *Descriptive analysis*: mapping of data sources to describe distribution of studies in terms of number of studies, methodologies, populations, sources and primary foci.
2. *Thematic analysis:* defining the primary and secondary themes in the literature, mapping them to the current research questions, and analysing the relevant findings. Thematic analysis was conducted using an inductive model in which the included data were analysed without a priori conceptions or specific hypotheses to test [24, 25].

The goal was to explore the nuanced issues related to riding a golf cart versus walking the course in a competitive environment, including inclusion, safety, performance and fairness, without testing a specific hypothesis. The process involved familiarisation with the data, generating initial codes, and allowing themes to emerge organically based on recurring patterns and insights.

## Results and Discussion

### Descriptive Analysis

A flow diagram following PRISMA-ScR conventions was created and reports the output from the study search and selection process (Fig. 1). The initial search identified 879 potentially eligible articles from Web of Science, Scopus, ProQuest Central and PubMed databases, and an examination of the citations of relevant articles. Of these, 299 were found to be duplicates and were removed. The abstracts of 580 articles were screened, with 457 not meeting the inclusion criteria. A full-text review was conducted on 123 eligible articles; 51 were excluded (47 because they did not meet inclusion criteria, and 4 because the full text was not available). Data extraction was conducted from 72 included articles, and consistent with scoping review methodologies no formal quality analysis was conducted [20–23] (Table 1 of the Electronic Supplementary Material [ESM]).

### Characteristics of Studies

Of the included studies, there were 39 (54.2%) articles relevant to Question 1 “*What are the main issues relating to inclusion and safety in golf?*”, 48 (66.7%) relevant to Question 2 “*What evidence exists regarding performance and competitive fairness in golf?*” and 14 (19.4%) articles being relevant to both questions. There were 20 (27.8%) literature review articles (including systematic, scoping and narrative reviews) [3, 4, 26–43]. Seventeen articles (23.6%) were cohort or case–control studies [6, 44–59]. There were ten (13.8%) official policy documents [12, 19, 60–67]. Nine articles (12.5%) were experimental studies [68–76], and six (8.3%) were legal case analyses [13, 14, 77–80]. Five articles (6.9%) were mixed or miscellaneous methods studies [81–85], and five (6.9%) were epidemiological studies [18, 86–89].

There were 55 (76.4%) articles that considered amateur golfers, 22 (30.6%) considered professional golfers [with 12 (16.7%) considering both] and eight (11.1%) that were conducted in non-golf contexts (e.g. articles that address relevant sports physiology or legal and ethical themes, but do not refer specifically to golf). Articles originated from 16 different nations: North America (USA: *n* = 33, Canada: *n* = 2); Europe (UK: *n* = 20, France: *n* = 2, Spain: *n* = 2, Sweden: *n* = 2, Austria: *n* = 1, Denmark: *n* = 1, Finland: *n* = 1, the Netherlands: *n* = 1, Switzerland: *n* = 1, Turkey: *n* = 1); Australia (*n* = 4); and Japan (*n* = 1). This distribution reflects wider trends in golf science [4]. All articles were published between 2000 and 2024 with the exception of two articles from 1994 and 1998, respectively, that were identified through citation searching.

### Thematic Analysis

#### Overview of Themes

The thematic analysis revealed seven key themes: physical demands of golf (*n* = 42), inclusion and accessibility (*n* = 24), cognitive demands of golf (*n* = 15), legal and ethical issues (*n* = 12), nutrition and hydration (*n* = 11), competitive fairness (*n* = 11), and safety and injury risk (*n* = 6). The majority of studies addressed two or more themes. A summary of the key components of each theme is provided in Table 1. In the thematic analysis, discussion regarding nutrition and hydration is integrated into the physical demands, cognitive demands and competitive fairness sections because of its close relevance to these themes.

**Table 1:** Summary of themes relating to the study questions

Theme	Number of studies (%)	Main findings
*Question 1: What are the main issues relating to inclusion and safety in golf?*		
Inclusion and accessibility	24 (32.9)	*Principle of inclusion*: golf aims to provide equitable participation for all, while recognising a need to support competitive integrity *Benefits of accessibility*: inclusion can improve mental health, well-being, physical health, self-worth/identity and community-building *Role of golf carts*: golf carts enhance accessibility, enabling participation by some players with impairments by modifying physical demands *Implementation challenges*: weather, inconsistent infrastructure, lack of guidelines, inadequate routes, regulations and cultural stigma limit golf cart availability at some clubs and venues *Legal support*: laws mandate reasonable accommodations to increase accessibility. The PGA Tour vs Casey Martin case set a precedent for supporting inclusivity relating to golf cart use where a competitive advantage for golf cart users cannot be demonstrated
Safety and injury risk	7 (9.6)	*Prevalence of golf cart injuries*: golf cart-related injuries are common, particularly in non-golf course settings, with older adults and adolescents being the most vulnerable groups. Participant injuries during elite/professional competitions are not common *Safety interventions*: speed limiters, seat belts, enhanced stability and guidelines can reduce the likelihood and severity of injuries *Safety in thermal stress*: golf carts may mitigate heat-related risks by reducing physical exertion, providing shade, and offering cooling effects during high-temperature events. Walking may mitigate cold-related risks during low temperatures
*Question 2: What evidence exists regarding performance and competitive fairness in golf?*		
Physical demands of golf	42 (57.5)	*Physical demands of golf*: golf requires moderate aerobic fitness, strength, balance and flexibility, with players covering over 7 km per competitive round and performing over 2000 swings during tournament weeks *Golf (walking) vs golf (using golf cart)*: walking the course while golfing increases energy expenditure compared with riding a golf cart. Walking is moderate-intensity activity (4.3–4.5 metabolic equivalents of task [METs]); golf cart use reduces physical intensity (3.5 METs), diminishing cardiovascular benefits. Based on 2024 Compendium of Physical Activity *Effects of golf carts*: golf carts reduce physical demand, and it is physiologically possible they could improve performance. They also limit thermal strain and minimise heat-related performance decrements *Age-related variability*: older golfers face greater metabolic challenges, with elderly players spending 70% of their round at or above 70% of the maximum heart rate, compared with younger players who are largely at low-to-moderate intensity *Tournament fatigue*: golfers experience cumulative fatigue, which may negatively affect motor coordination, shot accuracy and decision making, particularly in later stages of rounds, or tournaments *Hydration and nutrition*: dehydration and insufficient nutrition may impair endurance, cognitive performance and shot accuracy. Proper hydration and carbohydrate intake could mitigate these effects
Cognitive demands of golf	15 (20.5)	*Cognitive benefits of walking*: walking during recreational golf enhances mental sharpness, decision making and shot execution, particularly in older players, and supports broader cognitive functions such as memory, processing speed and executive function *Impact of fatigue and dehydration*: cognitive clarity may decline under fatigue and dehydration, with reduced decision making, shot accuracy and distance judgement linked to lower blood glucose and hydration during a round *Mitigation strategies*: limited evidence suggests carbohydrate replacement and hydration strategies help preserve cognitive and motor performance, reducing fatigue and sustaining shot accuracy *Fatigue and skill execution*: fatigue impacts shot consistency and putting accuracy, with physical and cognitive tasks showing performance degradation under stress *Cognitive preservation through golf carts*: by alleviating physical exertion, carts could help preserve decision making, reaction times and strategic planning, particularly in competitive contexts. They could also allow more time for mental preparation depending on if golf cart speed is limited
Nutrition and hydration	11 (15.1)	*Energy demands*: golf requires a mix of explosive and aerobic effort. Walking requires most of the 600–1400 kcal energy expenditure per round *Nutrition*: carbohydrate and protein intake before/during play may support recovery, focus, fatigue reduction and supports motor performance *Dehydration*: limited evidence suggests dehydration impairs endurance, cognition and shot accuracy; golf carts may reduce exertion and heat stress *Recovery aids*: protein, carbohydrates and hydration aid recovery; riding a golf cart may complement this by conserving energy *Golf cart vs walking*: golf carts reduce energy expenditure, heart rate and sweat loss, potentially aiding fatigue management and performance, especially in long tournaments and heat. Nutrition strategies are vital for walkers, while golf carts could reduce the need for careful energy management
Legal and ethical issues	11 (15.1)	*Inclusion vs competitive fairnes*s: legal precedents mandate reasonable adjustments for disabled athletes while seeking to preserve the essence of sport and the integrity of fair competition *Martin vs PGA Tour (2001)*: allowed Casey Martin to use a golf cart in elite competitions on the grounds of his disability, ruling that this accommodation enables equitable participation with the PGA Tour not able to demonstrate an impact on competitive fairness *CAS rulings*: deny aids that enhance performance beyond offsetting a disability, maintaining fairness in competitions *Golf cart policies*: governing bodies have produced policies that promote inclusion, with clear guidelines for golf cart use that aim to improve accessibility, implementation, compliance, and the retention of disabled and older golfers
Competitive fairness	10 (13.7)	*Fairness concerns*: golf carts reduce physical exertion, potentially giving users an advantage over walking players, particularly on challenging terrain, extended competitions or where high thermal strain is anticipated *Endurance advantage*: golf carts may help conserve energy and delay fatigue, while walking golfers may experience fatigue-driven declines in focus and accuracy *Disability considerations*: for players with higher energy costs because of conditions such as cerebral palsy, golf carts can restore fairness but require a careful assessment to ensure proportionality *Environmental conditions impact*: golf carts reduce strain under extreme conditions (e.g. heat, humidity, wind), offering cooling and protection that walking players lack; benefits must be regulated to avoid unfair advantages. Walking may be advantageous in cold conditions *Mitigation strategies*: proposals include restricting golf cart speed to walking pace, limiting environmental features such as a roof/windshield, and reserving golf cart use for eligible players with documented medical needs where providing a golf cart returns someone towards a level playing field but does not provide a competitive advantage
Knowledge gaps and research priorities	N/A	*Quantifying advantage*: more data are needed on how golf carts modulate physical and cognitive demands in high-level golfers, especially in varied environmental conditions (e.g. heat, hills) and varied athlete characteristics (sex, age, medical profile) and on golf measures such as strokes gained, driving distance and club head speed *Nutrition and hydration*: the effects of reduced physical exertion on energy and fluid requirements during golf cart use require further study *Proportionate accommodations*: more evidence needed to quantify performance limitations and altered physical/cognitive demands experienced by golfers with disability or medical conditions. This will guide the proportionate use of enabling measures *Interdisciplinary approach*: collaborative research across fields can ensure policies balance inclusivity and fairness effectively

*N/A* Not applicable, *PGA* Professional Golf Association

#### Inclusion and Accessibility

The principles of inclusion in golf aim to provide participation opportunities for all, including for individuals with disabilities. Bennett found that the benefits of widening access to golfers with disabilities include improved mental health, physical activity, identity reconstruction and community-building opportunities [85]. However, there are barriers to participation including social exclusion, cost, accessibility, lack of awareness and negative stereotypes. Golf carts significantly enhance accessibility for some by limiting the physical challenges associated with walking a golf course. For some individuals with a disability, golf carts serve as tools for autonomy, allowing them to participate in a sport that might otherwise be inaccessible because of the physical demands of walking long distances. However, the implementation of inclusive measures is often inconsistent across clubs, tournaments and national governing bodies.

Research underscores the importance of golf carts as adaptive tools for individuals with mobility impairments. Bennett and Monforte et al. identified transport solutions, including golf carts, as key to enabling some disabled players to access courses and participate in tournaments [43, 85]. A global study by Guillaume et al. involving 1734 disability golfers across 44 countries found that 63% of respondents required the use of a golf cart [18]. The necessity for golf carts varied by disability type, and when considering players with neurological, orthopaedic or amputation-related impairments, 20–27% of these players relied on riding golf carts for on-course mobility. For players with knee osteoarthritis, golf carts assisted participation in golf and permitted golfers to meet minimum physical activity recommendations, while reducing pain and surrogate markers of acute inflammatory response when compared with walking [58]. Specifically, golfer-reported knee pain was significantly higher at the completion of a walking round (mean pain increase 2.6 ± 2.4 points on a 0–10 pain scale) whereas no such change was reported when they completed a round using a golf cart (mean increase = 0.33 ± 1.41). The authors identified a significant increase in serum levels of inflammatory cytokines (tumour necrosis factor-α, interleukin-1β and interleukin-6) at halfway and round completion when walking but not with the golf cart.

Legal frameworks have promoted inclusivity in golf. The UK’s Equality Act 2010 and the US Americans with Disabilities Act mandate reasonable accommodations for disabled individuals in sports, which may include the provision of golf carts [11, 12]. An example of this was the US Supreme Court case involving professional golfer Casey Martin, who successfully petitioned to use a golf cart in PGA Tour tournaments because of a physical disability in the form of a rare circulatory condition [14]. This landmark case set a precedent requiring golf organisations to consider inclusivity along with competitive integrity.

Within the elite or professional context, leading golf organisations have worked to provide opportunities for those with a disability to compete. Organisations including the USGA, The R&A and the European Tour Group have worked to establish the US Adaptive Open, the G4D Open and the G4D Tour, with EDGA providing further competitive pathways and supporting recreational participation, enhancing physical and mental health outcomes [17]. These organisations have prioritised inclusivity by permitting adaptive measures such as golf carts for players who require them. However, despite the benefits of golf carts for accessibility, their implementation often faces challenges related to infrastructure and logistics. Some facilities lack infrastructure, clear guidelines for golf cart usage or appropriate routes, hindering accessibility [61, 62]. Both England Golf and Scottish Golf have provided practical guidance on facilitating inclusion, emphasising equal access for disabled players and the need for reasonable adaptations, including accessible routes and the use of golf carts under defined criteria such as ground and weather conditions [61, 62, 66]. Despite such policies, inconsistencies in implementation remain a significant challenge.

Golf carts are frequently stigmatised as tools of convenience for older or disabled players, reinforcing stereotypes that associate riding a golf cart with diminished athleticism [83]. This perception has created cultural tensions within the sport, where walking remains idealised as the traditional mode of play in some countries. Media representations often perpetuate these biases, portraying golf cart use as a deviation from the sport’s values of physical endurance and tradition. This stigma discourages broader use of golf carts in some cultures, even among players who might benefit from their availability. For instance, individuals recovering from injuries or dealing with temporary impairments may avoid golf carts because of perceived judgement limiting the social, health and well-being benefits golf can provide. Overcoming these cultural barriers requires an educational outreach to emphasise their role in promoting inclusivity in some contexts [83, 85]. In a competitive setting, many players and officials believe walking to be an integral part of the competition, although this view is not held universally.

#### Safety and Injury Risk

Riding a golf cart, while enhancing accessibility and convenience, can pose an increased safety risk in some contexts (e.g. from injuries associated with golf cart accidents). However, there is no convincing evidence of a significant safety risk for players in professional golf settings where health and safety provision can be carefully planned, noting that courses with uneven terrain or inadequate drainage systems may not always accommodate golf cart use without potential damage to the course or safety issues.

The existing literature focuses almost entirely on the use of golf carts in amateur recreational golf settings, and in the context of golf carts being used as transport on public streets or roads. Golf cart-related injuries are likely to be more common than many may think, with an analysis by McGwin et al. demonstrating 48,255 injuries between 2002 and 2005 in the USA, equating to 4.14 per 100,000 population [87]. Nearly half (45%) of golf cart injuries occur in recreational golf or other sporting environments, with older adults and adolescents being the most frequently affected groups. The US National Safety Council has estimated over 15,000 golf cart-related injuries annually, with 70% associated with use on ‘sporting facilities’. [86]. Head injuries and fractures are particularly prevalent, emphasising the vulnerability of these groups during golf cart use, although the vast majority of very serious incidents are away from the golf course. Castaldo et al. analysed 875 crashes over 8 years, highlighting the serious consequences of golf cart accidents [89].

Several studies recommend interventions to address the risks associated with golf cart use. Safety features such as health and safety briefings, speed limiters, seat belts and front-wheel brakes can significantly reduce the likelihood and severity of injuries [4, 86]. Safety briefings should be provided to all golf-cart operators when large spectator volumes are expected at events.

Where the use of golf carts may potentially improve safety, is in the context of events being played in very hot or humid ambient temperatures if they were available to all competitors or those with an enhanced risk of heat illness. Ahead of the 2024 Summer Olympic Games in Paris, Bandiera et al. considered the heat risk assumed by athletes in different disciplines [67]. Golf was categorised as a sport with a moderate risk of heat stress owing to its outdoor nature and long duration, reduced by the comparatively lower intensity of physical activity compared with high-intensity sports. Risk of heat stress could be mitigated by using golf carts through a number of mechanisms including the provision of shade and reduction in exercise intensity.

#### Physical Demands of Golf

Luscombe et al. performed a systematic review focusing on the physical activity demands of golf [3]. The authors concluded that, in general, golf can provide moderate-intensity physical activity taking place over an extended period of time. In a recent scoping review, O’Donnell et al. described that golf can also provide low-, moderate- or high-intensity aerobic exercise depending on the age and fitness of participants, the layout of the course and whether a golf cart is being ridden [33]. For older adults, it can provide extended periods of high aerobic intensity. In contrast, for younger fit persons, activity may be predominantly low intensity.

**Fig. 2:**
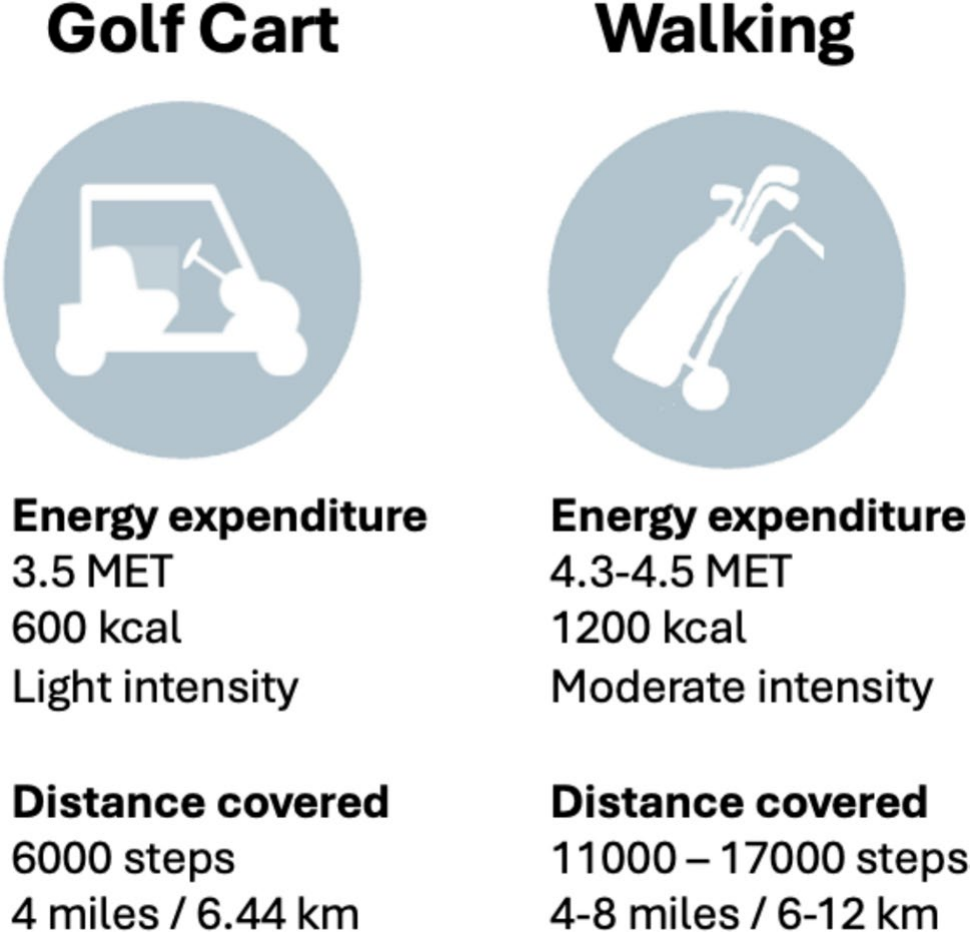
Summary of physiological demands associated with walking the course or using a golf cart for 18 holes. Adapted from Murray et al. (2017) and 2024 Compendium of Physical Activities [4, 28]. MET metabolic equivalent of task

There are some medical conditions and disabilities, where the resting metabolic rate and relative exercise intensity are higher than the general population. For example, individuals with cerebral palsy have been found to have a 22% higher resting metabolic rate after adjusting for fat-free mass, and have a significant increase of energy expenditure and oxygen cost during walking compared with age-matched peers [90, 91]. In general, the available evidence suggests that there is an increased physiological demand for those walking the course compared with those riding a golf cart [3, 4, 28, 33, 49]. This information is summarised in Fig. 2, which shows a higher MET, overall step count, energy expenditure and distance walked by those walking the course compared with those using a golf cart. The MET describes the body’s oxygen uptake for a given activity and is a measure of exercise intensity. The 2024 Compendium of Physical Activity demonstrates that walking the golf course while carrying clubs or pulling clubs in a trolley is associated with a MET of 4.3 and 4.5, respectively, whereas golfing while riding a golf cart has a lower MET of 3.5, corresponding to a lower oxygen demand and relative exercise intensity [28].

Regarding energy expenditure, Crowell found that those riding a golf cart had a significantly lower energy expenditure (5.2 kcal/min) than those pulling or pushing a hand trolley or carrying their clubs (6.8–7.5 kcal/min) [92]. Lyerly et al. demonstrated that walking nine holes required nearly double the calories of riding a golf cart (710 kcal vs 358 kcal) [54]. When considering distance covered during a round, a shorter distance is walked riding a golf cart compared with pulling or pushing a hand trolley or carrying clubs [3, 92]. In summary, despite methodological limitations and small numbers in many of the studies, evidence is generally consistent that riding a golf cart notably reduces physical activity attained in terms of METs, energy expenditure, heart rate and distance covered.

##### Physical Demands in Relation to Competitive Fairness

Aerobic fitness is an important element in competitive golf, and can be helpful for tournament golfers who often play many consecutive days. Zoffer in a narrative review observed that longer courses demand higher aerobic capacity, with fitter players demonstrating a slower onset of fatigue and better tolerance for heat stress [36]. McBride argued that fatigue accrued during multi-round tournaments can differentiate players, rewarding those who can minimise performance variability under physical and environmental stress [30]. Studies such as Forestier and Nougier and Mathers and Grealy found that fatigue adversely impacts motor coordination, with reduced accuracy in multi-joint tasks such as putting and swinging under fatigued conditions [52, 68]. Furthermore, Mathers and Grealy identified that golfers altered their scaling strategies (described by the authors as the strategies used by golfers to putt at different distances) under fatigue, leading to an inconsistent putting performance.

It is possible that golfers walking the course may experience greater cumulative fatigue, which can negatively affect performance, particularly in multi-round tournaments compared with those riding a golf cart. These players may need to prioritise more time for physical recovery, enabling less time for practice and other activities. O’Donnell et al. reported that energy expenditure during an 18-hole round is reported to range from 650 to 2000 kcal, and that walking between shots contributes the majority of this demand, but that studies that better evaluate energy expenditure are required [33]. The range in energy values can reflect differences in research methodology, individual physiology, course length, altitude, temperature, humidity and terrain. Fatigue may manifest as performance decrements in the latter stages of play. A review by Berlin et al. emphasised the broad physiological demands of golf, noting that players cover substantial distances per round, can perform over 2000 swings during a tournament, and rely on strength for driving distance and accuracy [29]. These demands place stress on all energy systems, requiring sustained low- to moderate-intensity aerobic effort with intermittent bursts of explosive power. Higdon reported that riding a golf cart mitigated fatigue, with mixed performance associations. This included reductions of 2–2.5% in clubhead velocity, but improvements of 7.1–9.4% in markers of biomechanical consistency at the top of the backswing and at contact [56]. Further golf shot data are required to evaluate these effects fully.

Environmental factors such as heat and humidity can amplify the physical demands of golf. Piil et al. highlighted the effects of thermal load on golfers, noting that even small increases in core body temperature (0.06–0.22 °C) could impair vigilance and dual-task motor-cognitive performance [81]. These conditions are particularly relevant in professional golf tournaments, where players may compete over several days in high temperatures. For example, at the 2023 FedEx St. Jude Championship in Memphis, temperatures reached 43.9 °C, and several players reported subjective performance decrements because of heat stress. Berlin et al. reported that dehydration could contribute to increases in core body temperature, and cardiovascular strain, which could impair endurance and cognitive performance [29]. Aylwin et al. suggested that riding a golf cart could mitigate these effects by reducing intrinsic heat generation and providing passive cooling benefits, such as shade [93]. In preparation for the golf competition at the Paris 2024 Olympic Games, Bandiera et al. (2024) categorised golf as a moderate-risk sport for heat stress due to long periods of activity and recommended strategies to mitigate the effects of heat that included the introduction of regular breaks, shade, cooling fans, and a focus on maintaining hydrated states [67]. Although golf carts were not suggested as a mechanism to reduce heat risk, their use could facilitate some of these potentially protective effects.

The Berlin et al. review also examined a number of studies that investigated the effect of hydration on performance. It surmised that dehydration greater than 1% bodyweight may have a negative impact on cognitive function and motor skills (affecting shot selection, accuracy and distance control), and dehydration > 2% body weight was associated with impaired endurance. However, the authors urged caution that a range of different methods have been used to assess hydration by existing studies, including a urine analysis (specific gravity and osmolality), plasma analysis (osmolality), bioelectric impedance, sweat rate and body weight, and argues that surrogate markers of hydration status have limited validity, and standardised objective measures should be used.

There are studies outlining that hydration and nutrition may play roles in mitigating fatigue-related declines in golf; however, many of the hydration studies use surrogate measures with limited validity. Smith et al. observed that dehydration impaired both cognitive and motor performance, including shot distance and accuracy [71]. They found that dehydration of greater than 2% body weight was associated with significant impairments in endurance and cognitive performance. Mild dehydration (1–2% body weight) negatively affected sport-specific cognitive abilities such as shot planning and execution and was associated with significantly impaired motor performance. Dehydrated golfers achieved poorer shot distance, with carry distances that were on average significantly short of the target, whereas the euhydrated group performed better. Further, the dehydrated group achieved poorer ‘on/off-line’ lateral accuracy (with a mean error of 7.9 m vs 4.1 m) on a task in which they executed shots using three different irons (aimed at simulated targets placed 110 m [9-iron], 125 m [7-iron] and 140 m [5-iron], respectively). The subjects also demonstrated poorer performance on simulated distance judgement tasks in which they were shown images of 30 real-world approach scenarios and required to estimate distances to the target (mean error of 8.9 m vs 4.2 m) [71]. Although these studies point towards hydration impacting performance, sample size and methods to calculate hydration mean these findings are presented with caution.

Further evidence is required to evaluate whether a reduction in energy expenditure or relative exercise intensity confers a competitive advantage in both amateur and professional players. Nevertheless, it is well understood that golfers experience physical and mental fatigue in the latter stages of a round, therefore it is reasonable to suggest that any such reduction may contribute to improvements in performance, recovery and reproducibility [33]. This may be complicated in the cases of players with disabilities (who may have higher baseline energy expenditure), and in some of these contexts the use of a golf cart may not confer a competitive advantage.

#### Cognitive Demands of Golf

Lorist et al. demonstrated the interaction between physical and cognitive fatigue, showing that performance on a cognitive test (choice reaction time) deteriorated significantly with increasing fatigue during tasks requiring concurrent physical and cognitive effort [70]. Fatigue-related declines in cognitive clarity have clear implications for skill execution in golf. Mathers and Grealy showed that moderate fatigue reduced the consistency of putting performance in six elite amateur golfers [52]. Specifically, fatigue altered their motor control strategies, leading to inconsistent execution even in successful putts.

Dehydration and heat stress may exacerbate cognitive fatigue and may impair decision making during golf. Smith et al. demonstrated that seven competitive amateur players who were subjected to 12 h fluid restriction, exhibited significantly impaired motor and cognitive performance [71]. Shot distance decreased (141 yards to 125 yards), off-target accuracy worsened (4–9 yards) and cognitive distance judgement errors also increased from 5 to 10 yards. These findings suggest that hydration deficits could degrade performance outcomes critical to golf. Environmental stressors, particularly heat, could further compound these effects [81]. Small rises in core temperature (0.06–0.22 °C) have been shown to degrade vigilance and dual-task performance. Strategies that reduce physical exertion and limit thermal strain, such as riding a golf cart, may help mitigate these performance losses. Wolkodoff et al. investigated a small cohort of amateur golfers in temperatures between 17.7 and 30 °C and found that subjective ratings of mental focus and performance outcomes, such as scores relative to par, were significantly better or trended toward improvement when golfers walked compared with riding a golf cart [49]. Specifically, golfers walking and using an electric trolley for clubs reported the highest mental focus score (6.6/10), followed by those walking using an unpowered trolley (5.7/10), with those using a golf cart reporting the lowest score (5/10). The players recorded scores that were, on average, one stroke lower in the walking conditions (either carrying or using a trolley) than the golf cart condition.

Walking the course during a round of golf is associated with several cognitive benefits, as sustained physical activity can enhance mental sharpness, decision making and shot execution. Kettinen et al. demonstrated improved cognitive performance (indicated by improvements in performance on a Trail Making Test) in older adults after walking a golf course, with the beneficial effects of golf being similar to that of Nordic walking [48]. The 2018 US Physical Activity Guidelines linked moderate-to-vigorous physical activity to improved cognitive performance, including faster processing speed, better memory and enhanced executive function [94]. The evidence was particularly compelling for older adults (aged > 50 years) compared with younger populations. Indeed, engagement in regular bouts of moderate or vigorous activity (in addition to resistance training and balance-promoting activities) forms the basis of the World Health Organization’s guidelines on physical activity [95]. Thus, walking while playing golf may confer a dual benefit of maintaining physical activity while enhancing cognitive engagement, particularly in older golfers. Conversely, the cognitive benefits of walking, fatigue and dehydration introduce trade-offs that could diminish cognitive clarity, particularly in challenging environmental conditions or during the later stages of a round, and indeed any multi-day event.

It is plausible that riding golf carts may help preserve cognitive function by reducing the physical fatigue associated with walking in multi-day competitions, on unusually hilly and or long courses, or in hot and humid conditions. By alleviating physical exertion and thermal strain, it is feasible that riding a golf cart may enable players to maintain sharper decision making, and strategic planning in some competitive contexts. Smith suggested that minimising physiological fatigue allows golfers to maintain an optimal mental and technical state, thereby improving shot consistency and overall performance [41]. In addition, golf carts may allow for golfers to arrive at the ball earlier, and in these situations afford more time for preparation between shots. Overall, the evidence is that most of the time, physical activity associated with walking may be beneficial compared with riding a golf cart, but that towards the end of rounds and in some circumstances (multi-day competition, hot or humid conditions, or where dehydration and fatigue are likely), then it is biologically plausible that those riding a golf cart may have a competitive advantage.

#### Competitive Fairness and Mitigation of Potential Advantages

The use of golf carts in competitive golf raises issues regarding competitive fairness, primarily because of their effect on the physical and cognitive demands of the sport. As discussed in this scoping review, golf carts reduce physical demands by limiting the need to walk between shots, potentially providing an advantage to players who are permitted to use them compared with those who must walk. Any advantage would most likely be evident in challenging terrain, multi-day or multiple rounds per day competitions, and environmental conditions with a high thermal strain. Golf carts could offer an endurance advantage, as golf cart users limit fatigue-related declines that walking players may experience. Golf carts might enable players to sustain motor performance.

Evidence relating to the effects of golf cart use on cognitive performance is more mixed, with Wolkodoff et al. and Kettinen et al. noting improved cognition in amateur golfers walking the course, while other studies discuss potential advantages associated with the mitigation of fatigue, dehydration, energy expenditure and environmental effects—all of which could be achieved by riding a golf cart [41, 48, 49, 52, 70, 71]. Golf carts may allow golfers to maintain higher levels of execution late in a round [41]. By alleviating fatigue, golf carts may decrease the challenge of sustaining performance over time for those participants permitted to ride a golf cart. Walking-induced fatigue, particularly on long or hilly courses, can be significant and has been shown to degrade motor skills and shot consistency [30, 71].

For athletes with medical conditions associated with higher relative energy expenditure (e.g. those with cerebral palsy), golf cart use could potentially offset physiological disadvantages and restore fairness [90, 91]. However, the physical manifestations of such conditions present across a broad spectrum of severity, and athletes that are able to compete at high levels will likely demonstrate milder forms of disability. As a result, the appropriateness of enabling measures such as technology, golf carts and adjusted competition rules will be contingent on the proportionality of such interventions in the case of each individual’s requirements. The competitive implications of golf cart use are exacerbated under adverse environmental conditions. In extreme heat and/or humidity, the physical demands of walking increase because of an elevated core body temperature, potential dehydration and cardiovascular strain. Cheuvront and Kenefick and Piil et al. both highlighted that heat stress can degrade motor-cognitive performance [34, 81]. Riding a golf cart mitigates some of these effects by reducing intrinsic heat production from physical exertion and providing passive cooling benefits, particularly when golf carts include features such as roofs, windshields or air conditioning. Golf carts with a roof or windshield also provide protection in wet or windy conditions, reducing physiological strain compared with walking players who are more exposed to the elements. In cold conditions, being required to ride a golf cart may create an increased physiological demand to keep warm, compared with those walking due to decreased thermogenesis.

To address concerns regarding fair competition, while prioritising inclusivity for players with medical conditions, several mitigation strategies are feasible. Golf carts could be restricted to normal walking speed to prevent users from arriving at their shots significantly earlier, ensuring no additional advantage in strategic planning. Environmental features that provide protective benefits such as roofs, windshields or air conditioning should be limited, although practical alternatives such as umbrellas for shade could be permitted. Eligibility for golf cart use should either be for all players, or be confined to players with a qualifying medical condition, ensuring that golf carts act as a reasonable adjustment to offset physiological disadvantages without becoming a performance aid. Additionally, golf cart use should be restricted to the eligible player, excluding caddies or support staff (including coaches), to maintain alignment with competition requirements. An alternative may be to permit riding a golf cart for all participants, noting that this is not feasible at all golf facilities or tournament venues.

Cultural and psychological perceptions also pose challenges. Walking, is for many, ingrained in the tradition and challenge of professional golf, and the use of golf carts may be viewed by some as compromising the athletic challenge of the sport. This perception could lead to psychological discomfort for players and create stigma around golf cart use, or could provide a boost if they believe it confers an advantage. As a summary, Table 2 provides the key considerations regarding competitive fairness in elite competitive golf that may need to be considered.

**Table 2:** Key considerations regarding competitive fairness in elite competitive golf

Key questions	Current evidence and considerations
*Question 1*	
Is there a difference in physiological demands between riding a golf cart vs walking the course while playing golf?	There is compelling evidence of a difference in physiological demands between riding a golf cart and walking the course although more research is required using valid methodologies in high-standard golfers to fully explore this difference Those riding a golf cart have a decreased metabolic equivalent of task, energy expenditure, step count, distance walked and energy cost of playing golf It is physiologically likely, but not proven beyond reasonable doubt, that the decreased physical demands of riding a golf cart may impact competitive fairness in some circumstances Any competitive advantage may depend on factors such as course topography, fitness of the participant, weather conditions and volume of golf (e.g. multiple rounds on 1 or consecutive days) A golf cart may offset a disadvantage (as opposed to offering a competitive advantage) for participants with underlying conditions associated with increased energy expenditure. Assessment on an individual basis would likely be necessary in these cases
*Question 2*	
Is there a difference in cognitive demand/performance between riding a golf cart vs walking the course while playing golf?	There is a potential for competitive fairness to be impacted if there is a difference in cognitive demand and/or cognitive performance when riding a golf cart compared to walking the course or vice-versa There is some evidence at the recreational level of improved cognitive performance after walking/walking a golf course, but without direct comparison to a golfer riding a golf cart Conversely, there is some evidence as well as reasonable biological plausibility that using a golf cart while playing golf may reduce the negative effects of fatigue, potentially improving performance in some circumstances In competitive golf, the use of a golf cart might provide an advantage by enabling a golfer to arrive at their next shot earlier, thus having more time to prepare and execute a shot, reduce time pressure and lessen cognitive demand. Further, having the choice to switch between walking and riding a golf cart may provide a psychological advantage Overall, there is no compelling evidence of altered cognitive performance or demand when an individual either walks the course or rides a golf cart
*Question 3*	
Is there a difference in response to environmental conditions between riding a golf cart vs walking the course while playing golf?	Environmental factors such as air temperature, humidity, radiant heat and air velocity (wind) may affect performance and can also affect health. Even small rises in core body temperature (0.06–0.22 °C) may result in decreased vigilance and dual task performance Riding a golf cart compared to walking the course could alter a golfer’s physiological response to environmental conditions and potentially affect performance. Potential mechanisms include decreased energy requirements (leading to less intrinsic heat generation), and protection from the elements from any roof, windshield or other feature of the golf cart In very hot/humid conditions, it is more likely than not that decreasing thermal strain through use of a golf cart may confer a performance advantage. Conversely, in cold conditions, it is likely that decreased thermogenesis in golfers compelled to ride a cart may confer a performance disadvantage More original research in golf-specific contexts is required to evaluate thermogenesis and performance in hot and humid conditions
*Question 4*	
Does the best available evidence suggest a competitive advantage for persons riding a golf cart compared to walking in elite competition?	It is more likely than not that those permitted to ride a golf cart may gain a competitive advantage in some circumstances There is compelling evidence of a decrease in physiological demand between riding a golf cart and walking the course It is likely that in certain environmental conditions (e.g. hot and/or humid conditions, or hilly courses) that riding a golf cart could confer a competitive advantage, while in cold conditions being required to ride a golf cart may confer a disadvantage There is no compelling evidence of altered cognitive performance or demand when an individual either walks the course or rides a golf cart
*Question 5*	
Can any potential competitive advantage be eliminated or mitigated?	Some potential competitive advantages could be mitigated, for example by limiting any shade or protection from the elements, and limiting the golf cart speed to walking pace. Eliminating any potential advantage to support staff could be achieved by not permitting caddies/other support staff to use a golf cart or to transport clubs on a golf cart Other advantages may not be eliminated, such as the lower physiological demands associated with golf cart riding vs walking There are some medical conditions (e.g. cerebral palsy) associated with an increased resting metabolic rate and wider increased metabolic demands. It is likely in these circumstances that any potential advantage is likely to be mitigated or eliminated depending on the specifics of the individual case Any potential advantage could also be eliminated by permitting golf cart use to all participants. Persons permitted to ride a golf cart should be required to use these consistently, as opposed to be able to change based on environmental conditions and other factors The golf handicap system may support fair competition at the non-elite level
*Question 6*	
Do golf organisations consider inclusion or fairness a higher priority if a competitive advantage cannot be eliminated?	Policies vary between professional tour organisations, which may be partly based on priorities of the organisation and its members, as well as legal and operational considerations Some organisations support modified versions of golf competition, e.g. the G4D Tour, which provide opportunities for those with a disability to ride a golf cart while competing in G4D events
*Question 7*	
Are there age group considerations relevant to the use of a golf cart?	Senior/masters level competition (i.e. with participants over 50 years of age) may have an increased number of participants with a qualifying disability If physical fitness is viewed as an important component of golf, the decreased physiological demands and decreased thermal strain associated with riding a golf cart may offer an advantage to older players because fitness, recovery potential and resistance to the effects of hot/humid conditions decrease with increasing age

*G4D* Golf 4 Disabled

#### Legal and Ethical Considerations

The ethical issue of balancing inclusion with competitive fairness has been a long-standing discussion in elite sport, including golf. Legal decisions have set precedents for the allowance of mechanical aids or reasonable adjustments that aim to offset disadvantages caused by a disability without providing an overall competitive advantage. The landmark Martin versus PGA Tour case in the US Supreme Court (2001), established that reasonable adjustments, such as the use of golf carts as medical transport, could be permitted in elite golf [14, 77]. The court ruled that allowing Casey Martin to use a golf cart did not fundamentally alter the nature of the competition, as it simply addressed his mobility impairment caused by a circulatory condition. This decision underpinned the principle that accommodations should allow individuals with disabilities to compete equitably, without enhancing performance beyond the natural ability of the athlete. At this time, the PGA Tour could not demonstrate a competitive advantage through riding a golf cart, so inclusion was prioritised without, in the view of the court impacting competitive fairness.

Other conclusions have been drawn regarding the use of aids that may provide a performance-enhancing effect. The Court of Arbitration for Sport established that mechanical aids or adjustments could be denied if they provided an athlete with a performance advantage exceeding what is necessary to offset a disability. Specifically, the Court of Arbitration for Sport ruled that an athlete using a mechanical aid that enhances their performance beyond their inherent capacity, had they not had a disability, could be excluded to preserve competitive fairness [96]. Similar rulings have occurred in cases involving Therapeutic Use Exemptions for beta-blockers in precision sports [97], where the treatment was acknowledged to be medically required, but there were concerns regarding competitive fairness. Transgender participation has also been the subject of scientific, ethical and legal discussion regarding the fundamental principles of inclusion and competitive fairness. The global governing bodies (The R&A and the USGA) recently made clear in their 2024 policies that while they aim to prioritise inclusion, they have excluded participation in the female category for transgender female individuals who had gone through any part of male puberty. This is because of the competitive advantages male puberty can confer, including higher lean muscle mass, larger skeleton, greater cardiorespiratory capacity and benefits from training in a male sport context [9, 10].

These cases are relevant to golf, where there is no doubt that permitting riding a golf cart may maximise inclusivity, but it may provide a competitive advantage to those riding a golf cart in some circumstances. The Equality Act 2010 in the UK serves as a foundational legal framework that requires inclusive practices in sports, including golf. It follows that golf clubs must support accessibility for all players, providing reasonable adjustments, where necessary. England Golf’s policies operationalise these principles by offering clear guidance to clubs on implementing inclusive golf cart policies [61, 62]. They outline several benefits of doing so, including: compliance with legal requirements, positive action toward inclusion for members and visitors, reduced liability risks, and increased participation and retention of older and disabled players.

## Limitations

This scoping review has several limitations which should be acknowledged. First, the search was restricted to articles written or translated into English, which may have excluded relevant studies published in other languages. This limitation could partly explain the predominance of studies from English-speaking countries, such as the USA, UK and South Africa, in the included literature. Second, the review focused on articles published within the last 25 years to ensure the inclusion of contemporary evidence. While this approach prioritises up-to-date evidence, it may have excluded older studies with historical or foundational insights relevant to the topic. In accordance with scoping review methodology, the included studies were not subjected to a formal quality assessment. Although this approach allows for a comprehensive mapping of the literature, it limits the ability to evaluate the quality and reliability of individual studies against a consistent framework. Third, the studies included for review were concerned with all levels of golf (rather than purely competitive golf) because of the limited number of studies conducted in a competitive setting. Finally, the heterogeneity in study designs, methodologies and populations across the included articles presented challenges in synthesising the results. While this diversity reflects the multi-faceted nature of the topic, it also complicates direct comparisons and limits the generalisability of specific findings to all competitive golf contexts.

## Knowledge Gaps and Research Priorities

This review highlights important knowledge gaps related to the use of golf carts in competitive golf, particularly in the areas of inclusion, safety, physical and cognitive demands, nutrition, legal frameworks and competitive fairness (Table 3). Addressing these gaps is essential to inform policy decisions and promote both inclusivity and fairness in the sport.

**Table 3:** Key research priorities regarding potential competitive advantages for those riding a golf cart

Key questions	Research priorities
Are golf carts available and accessible to all golfers?	Understand whether golf carts are available to support play by those who require them at venues and in all conditions
What are the specific differences in physiological demands between riding a golf cart vs walking the course while playing elite golf?	Evaluate the nature and magnitude of differences in physiological demands in elite competitive players walking vs riding a golf cart in competition settings using validated methodologies Evaluate key performance metrics in elite golfers in each transport condition. This could include: shots gained, carry distance, shot dispersion, club head speed and counter movement jump. Assessments should consider temperate and hot/humid conditions separately
Is there a difference in cognitive demand/cognitive performance between riding a golf cart vs walking the course while playing golf?	Evaluate the change in cognitive load and performance between riding a golf cart and walking the course in amateur and professional contexts Qualitative research should explore insights from players, caddies, scientists and officials. Quantitative research should include at least: decision making and strategy formation, distance judgement, topographical judgement and mental fatigue
Is there a difference in response to environmental conditions between riding a golf cart vs walking the course while playing golf?	Original research in golf-specific contexts should evaluate thermogenesis and performance in hot/humid conditions Evaluate, through survey and interview-based research conducted with elite players and caddies, whether the use of a golf cart may alter physiological and psychological responses to environmental conditions, and potentially affect performance
Can any potential competitive advantage be eliminated or mitigated?	Strategies with the potential to eliminate/mitigate the potential advantages/disadvantages conferred by golf cart use should be evaluated in appropriate settings

## Conclusions

This scoping review identified 72 studies relevant to riding golf carts as on-course transportation in competitive golf. Golf carts play a crucial role in promoting accessibility, particularly for individuals with disabilities, and this is consistent with legal frameworks that require reasonable accommodations to increase inclusivity. There are however inconsistencies in implementation and sociocultural barriers to widespread adoption. There are also safety concerns, mainly when golf carts are not used on a golf course, relating to golf cart-related injuries and factors to mitigate this risk have been suggested.

In-round riding of golf carts is likely to reduce the physical demands, fatigue and environmental challenges of competitive golf. This may be performance enabling for golfers with disability-related limitations (i.e. mitigating those limitations to bring them up to a normal unencumbered baseline) but would likely be performance enhancing (i.e. confer an above-baseline competitive advantage) over golfers that walk the course in some circumstances. This has not been fully explored in high-quality research studies. Strategies to mitigate undesired performance-enhancing effects might include speed restrictions, design considerations, and careful thought regarding the balance between inclusivity and competitive integrity.

More research is needed to guide the fair use of this enabling technology within clearly defined contexts by: (1) evaluating the specific performance deficits associated with medical issues in which the use of golf carts might be considered permissible, and (2) evaluate the magnitude and nature of competitive advantages experienced by golf cart users.

## Supplementary Information

Below is the link to the electronic supplementary material.

Supplementary file1 (XLSX 46 KB)

## Declarations

### Availability of data and material

All data are publicly available in the published literature.
